# Reconciling multiple connectivity-based systems biology methods for drug repurposing

**DOI:** 10.1093/bib/bbaf387

**Published:** 2025-07-30

**Authors:** Catalina Gonzalez Gomez, Manuel Rosa-Calatrava, Julien Fouret

**Affiliations:** CIRI, Centre International de Recherche en Infectiologie, Team VirPath, Inserm U1111, Université Claude Bernard Lyon 1, CNRS UMR5308, ENS de Lyon, 8 rue Guillaume Paradin Faculté de Médecine RTH Laennec, Lyon 69008, France; International Associated Laboratory RespiVir France - Canada, Centre de Recherche en Infectiologie, Faculté de Médecine RTH Laennec, Université Claude Bernard Lyon 1, Université de Lyon, INSERM, CNRS, ENS de Lyon, Lyon 69008 France, Centre Hospitalier Universitaire de Québec - Université Laval, Québec, QC G1V 4G2, Canada; Nexomis, Université Claude Bernard Lyon 1, 8 rue Guillaume Paradin Faculté de Médecine RTH Laennec, Lyon 69008, France; CIRI, Centre International de Recherche en Infectiologie, Team VirPath, Inserm U1111, Université Claude Bernard Lyon 1, CNRS UMR5308, ENS de Lyon, 8 rue Guillaume Paradin Faculté de Médecine RTH Laennec, Lyon 69008, France; International Associated Laboratory RespiVir France - Canada, Centre de Recherche en Infectiologie, Faculté de Médecine RTH Laennec, Université Claude Bernard Lyon 1, Université de Lyon, INSERM, CNRS, ENS de Lyon, Lyon 69008 France, Centre Hospitalier Universitaire de Québec - Université Laval, Québec, QC G1V 4G2, Canada; Nexomis, Université Claude Bernard Lyon 1, 8 rue Guillaume Paradin Faculté de Médecine RTH Laennec, Lyon 69008, France; VirNext, 8 rue Guillaume Paradin Faculté de Médecine RTH Laennec, Université Claude Bernard Lyon 1, Université de Lyon, Lyon 69008, France; CIRI, Centre International de Recherche en Infectiologie, Team VirPath, Inserm U1111, Université Claude Bernard Lyon 1, CNRS UMR5308, ENS de Lyon, 8 rue Guillaume Paradin Faculté de Médecine RTH Laennec, Lyon 69008, France; International Associated Laboratory RespiVir France - Canada, Centre de Recherche en Infectiologie, Faculté de Médecine RTH Laennec, Université Claude Bernard Lyon 1, Université de Lyon, INSERM, CNRS, ENS de Lyon, Lyon 69008 France, Centre Hospitalier Universitaire de Québec - Université Laval, Québec, QC G1V 4G2, Canada; Nexomis, Université Claude Bernard Lyon 1, 8 rue Guillaume Paradin Faculté de Médecine RTH Laennec, Lyon 69008, France

**Keywords:** connectivity score, system biology, drug repurposing, differential expression signature, data integration

## Abstract

In the last two decades, numerous *in silico* methods have been developed for drug repurposing, to accelerate and reduce the risks about early drug development. Particularly, following Connectivity Map, dozens of distinct data-driven methods have been implemented to find candidates from the comparison of differential transcriptomic signatures. Interestingly, there have been multiple proposals to integrate available knowledge using systems biology databases and adapted algorithms from the network biology research field. Despite their similarities, these methods have been formulated inconsistently over the years, even if some of them are fundamentally similar. The aim of this review is to reconcile these integrative methods, focusing on elucidating their common structures while underlining the specificities of their strategies. To achieve this, we classified those methods into two main categories, provided schematic workflow representations, and presented a homogenized formulation for each.

## Teaser

- This review brings together twelve *in silico* methods for drug repurposing that integrate systems biology data, organizing them into two main categories: gene set-driven and network-driven approaches.
- Gene set-driven methods compare disease and drug transcriptomic signatures by mapping them onto predefined or data-driven gene sets, employing enrichment analyses or feature-space projections to quantify similarity or opposition.
- Across gene set-driven methods, there three main workflow steps are described: (i) identification of relevant gene sets or modules, (ii) selection of gene sets of interest based on the query signature, and (iii) calculation of modular or global connectivity scores to rank potential therapeutic candidates.
- Network-driven methods leverage biological interaction networks and network biology algorithms (such as propagation or shortest-path analysis) to reveal deeper, system-level relationships between disease and drug perturbations.

## Introduction

Drug development is a costly and time-consuming process, with an average cost that ranges between less than $1 billion and more than $2 billion per drug [1, 2] and takes between 5 and 20 years [3]. In addition, the field suffers from low success rates, with only 12% of molecules achieving FDA approval after entering the clinical stage [1]. To accelerate the discovery phase of drug development and to diminish the associated risks, repurposing (or repositioning) has emerged as a promising strategy to identify new therapeutic applications for existing drugs [4, 5]. To date, the majority of successfully repurposed drugs have been identified by retrospective clinical or pharmacological analysis [6]. However, there are ongoing efforts to develop systematic *in silico* approaches [7, 8]. In this review we focused on *in silico* repurposing strategy matching disease and drug omics signatures while integrating knowledge leveraging systems biology and network biology methods. The past two decades has witnessed the rise of signature-based approaches focusing on gene expression pattern comparison [9, 10]. Unlike target-based repositioning methods, which identify molecules that interact with a known target protein to provide a new indication [11], signature-based methods are target-agnostic and rely on the principle of polypharmacology [12, 13]. A significant advancement came with Lamb et al.’s development of the Connectivity Map (CMap) [9]. CMap screens disease (or drug) signatures against its drug-profiles database using a Kolmogorov–Smirnov (KS) based connectivity score. Alternative screening methods were derived from this principle with new connectivity scores [9, 10, 14] and similarity metrics [15] which all have been reconciled in the review from Samart et al. [16]. However, these signature-based methods have limitations: they cannot identify relationships without common genes and may overlook underlying gene relationships. Therefore, new methods relying on available knowledge have been developed integrating systems biology databases and network biology algorithms.

Knowledge-centered approaches, leveraging network biology, are widely used at drug discovery stage [17]. A fundamental method is the enrichment analysis [18] using functional genomics databases such KEGG [19] or GO [20] to identify significant pathways from disease-specific genes (e.g. deregulated genes). Enrichment methods range from over-representation analysis (ORA) [18] to more sophisticated approaches such as GSEA [21] or the parent–child method, which is based on the hierarchical structure of functional genomics databases [22]. Even more complex strategies leverage the topological aspects of functional genomics databases [23] including relationships between genes and/or other entities (e.g. metabolites or drugs), as well as causal inference [24, 25]. In the field of drug development, these approaches have been primarily used during the discovery phase for target identification [17, 26–28] and mechanism elucidation [29, 30].

Omics’ signatures comparisons while integrating available knowledge has been challenging. However, recent developments in network biology algorithms have facilitated the process [31] through specialized algorithms such as network propagation [32]. In this review, we aim to systematically analyze *in silico* repurposing strategies leveraging both the data-driven aspects by comparing disease and drug(s) omics signatures with the integration of available knowledge using network biology algorithms. We deliberately exclude chemoinformatics and molecular docking strategies, which typically intervene downstream in the drug development pipeline after target identification. Instead, we focused on computational methods that leverage disease signatures and network-based analyses to generate initial drug-disease or target-disease hypotheses, which can later be refined through structure-based approaches. We retrieved twelve published methods that, despite their similarities, often employ inconsistent terminology and notation. Inspired by Samart et al.’s [16], we propose a unified framework that homogenizes notation and identifies standard workflow steps, aiming to facilitate method comparison and guide future developments in systems biology-based connectivity estimation and drug repositioning.

## Methods taxonomy and general notations

**Figure 1:**
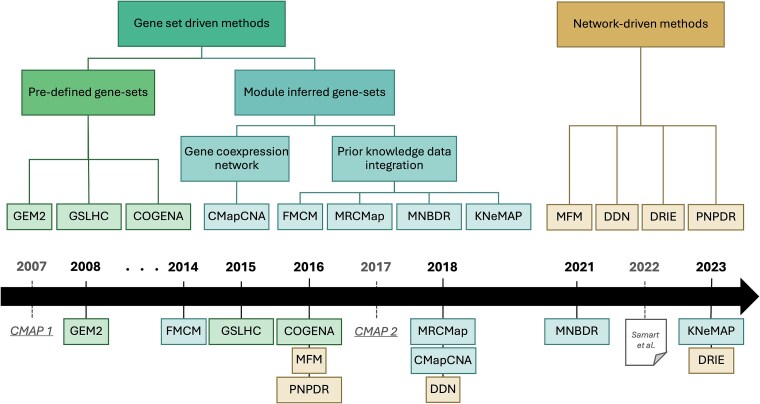
Taxonomy and chronology of system biology-based connectivity scores. The twelve scores were primarily classified depending on the mathematical object used to integrate systems biology knowledge into connectivity computations: Gene sets or networks. Among gene set driven methods, we distinguish predefined gene sets such as KEGG [19] or GO [20] with inferred gene sets using either data (gene co-expression) or prior knowledge. CMAP1 and CMAP2 connectivity scores were included in the chronology as references, along with the reconciling review by Samart et al. [16].

We performed a taxonomic classification of methods that compare omics signatures of two given perturbagens (e.g. drug and disease) while integrating systems biology data. These methods, listed in Table 1, fall into two main categories, as represented in Fig. 1. On one hand we have a set of methods relying on gene sets and on the other hand we have methods using biological networks as a graph. General notation used across methods are summarized in Table 2 [33–44].

**Table 1:** Methods names and abbreviations.

**Name**	**Abbreviation**	**Reference**
Gene Expression Modules-Based Similarity Search	GEMS2	[33]
Gene set Local Hierarchical Clustering	GSLHC	[34]
Co-expressed gene set enrichment analysis	Cogena	[35]
Functional Module Connectivity Map	FMCM	[36]
Master Regulators Connectivity Map	MRCMap	[37]
Connectivity Map Co-expression Network Analysis	CMapCNA	[38]^1^
Module Network Based Method for Drug Repositioning	MNBDR	[39]
Network mapping approach for knowledge-driven comparison of transcriptomic profiles	KNeMAP	[40]
Method of functional modules	MFM	[41]
Pathway-based Network Propagation for Drug repositioning	PNPDR	[44]^1^
DrugDiseaseNet	DDN	[42]
Drug Repurposing method based on the Inhibition Effect on gene regulatory network	DRIE	[43]

**Table 2:** General notation.

**Notation**	**Description**
*R*	The full set of genes of the reference perturbagen profile.
*R_X_*	Subset of reference genes with the most extreme scores, defined by fold-change and/or significance $\left({R}_X\subseteq R\right)$.
${R}_X^{+},{R}_X^{-}$	The up-regulated and down-regulated subsets of R_X_, the genes with the extreme perturbagen gene scores (${R}_X^{+}\cup{R}_X^{-}={R}_X$)
*S*	Full set of genes, or those selected based on prior knowledge (e.g. mutated genes), associated with the query perturbagen state.
*S_X_*	Subset of query genes with the most extreme scores, defined by fold-change and/or significance $\left({S}_X\subseteq S\right)$.
${S}_X^{+},{S}_X^{-}$	The up-regulated and down-regulated subsets of S_X_, the genes with the extreme perturbagen gene scores (${S}_X^{+}\cup{S}_X^{-}={S}_X$).
${v}_{ref}\left(\right),{v}_{qry}\left(\right)$	score function for the reference or the query signature that takes one or more genes as input and returns a vector of their gene expression scores.
$\Delta{v}_{ref}\left(\right),\Delta{v}_{qry}\left(\right)$	score function for the reference or the query signature that takes one or more genes as input and returns a vector of the signed normalized measured of their gene expression changes (e.g. log fold change).
*N_R_, N_Rx_, N_S_, N_Sx_*	Number of genes in *R, R_X_*, *S* and *S_X_*.
GeneSetDB	Database with the predefined gene sets. Commonly, those sets represent a group of functionally associated genes (e.g. GO, MSigDB, KEGG), that act in concert to carry out a specific function.
RefProfDB	Database with the gene expression profiles used as reference for the similarity search (e.g. Connectivity Map database).
*N_refDB_*	Number of reference profiles in the RefProfDB.
$r$	Reference instance in the RefProfDB $r\in [\![ 1,{N}_{refDB}]\!]$.
$n$	Number of enriched gene sets in the query (or identified gene modules).
$i$	Gene sets, or gene modules, instance $i\in [\![ 1,n]\!] .$
${gm}_i$	A gene module instance, i.e. a collection of genes in the i-th gene module.
$\rho$	Pearson’s correlation coefficient.

## Gene set driven approaches

### Generalized approach for gene set driven methods

To generalize all those methods (see Fig. 1) into a general framework, we have named three recurrent steps. First (i) the identification of gene sets, then (ii) the selection/extraction of those gene sets using the query signature and finally (iii) the calculation of the score that can be modular (at the gene set level) and/or summarized. Below, we shortly describe the principles of those steps across the different methodologies before presenting each method using a common terminology.

### Gene sets identification

**Figure 2:**
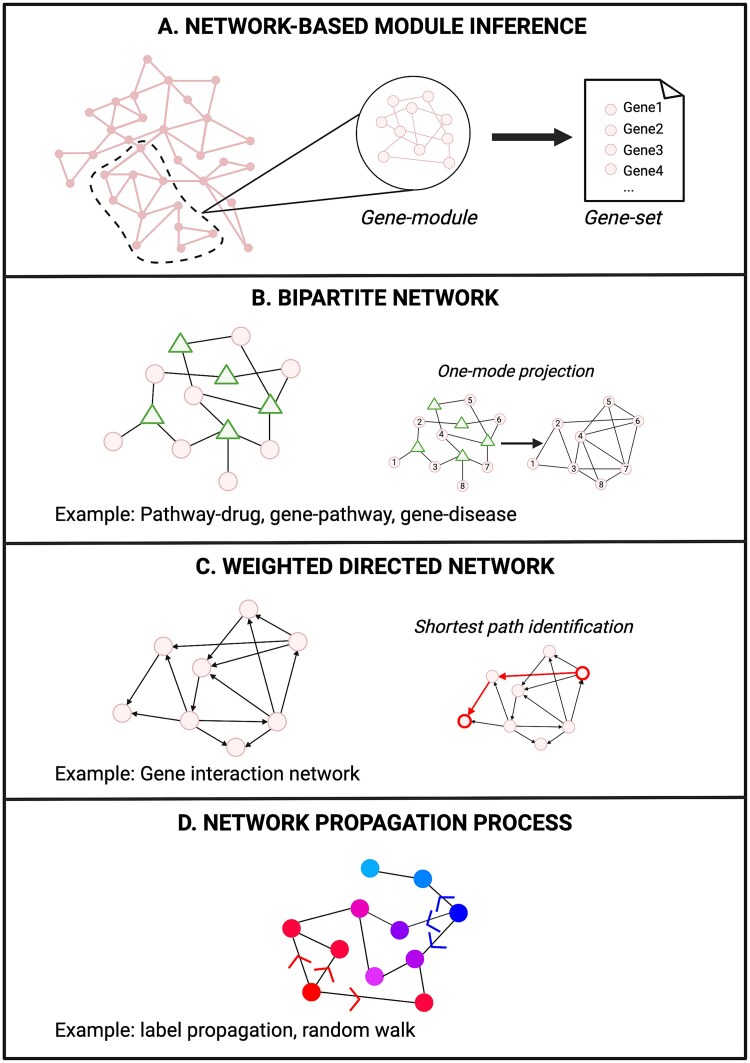
Schematic representation of key concepts in network analysis. (A) Network-based module inference. Different clustering strategies exist, such as agglomerative hierarchical clustering, to identify communities or modules of related nodes. (B) Bipartite network. These networks are characterized for having two classes of nodes with edges only existing between nodes of different classes. They can be transformed by suppressing one class and connecting nodes with common neighbors. (C) Weighted directed network. In this type of network, each edge has an associated direction and a weight. The shortest path between two nodes can be identified by finding the path that minimizes the sum of weights. (D) Network propagation process. This process can be applied to different types of networks as a way to spread information across them. Created in BioRender. Gonzalez Gomez, C. (2025) https://BioRender.com/h78r633.

The aim of this step is to find one or many gene sets. GEMS2 [33] and GLHSC [34] directly extract functional gene sets from predefined databases such as KEGG [19], GO [20] or MSigDB [45, 46]. Other approaches, like FMCM [36], MRCmap [37], CMapCNA [38], MNBDR [39], and KNeMAP [40] build gene sets from data-inferred networks in combination with module identification algorithms as depicted on Fig. *2*A. Each method proposes its own network construction strategy, from prior-knowledge data and/or experimental data. A bit apart, Cogena [35] uses network-free hierarchical clustering from experimental data to identify gene sets while annotating them with functional databases (e.g. GO).

### Selection of gene sets of interest

The aim of this step is to ensure that only the most pertinent gene sets are considered for scoring. GEMS2, GLHSC, and MRCMap use enrichment analysis (ORA or GSEA) to determine the enriched gene sets. MNBDR maps the query signature on the network and leverages the topology to select relevant modules. FMCM uses ORA to select enriched functional modules and then finds key genes within them based on network topology. Cogena and CMapCNA bypass the selection phase by directly using query signature to infer the gene sets through clustering or network construction followed by gene module identification. Of note, KNeMAP retains all identified gene sets.

### Scores calculation

Finally, the selected gene sets are used to calculate the scores between the reference profiles and the query signatures. Scoring strategies vary, either focusing on individual gene sets (modular) or considering the entire profiles (global).

#### Modular scores

Some methods calculate a score between each gene set of interest and each reference profile. Cogena uses an enrichment score to compare gene sets inferred from query clusters and references differential expression genes (DEG). GEMS2, FMCM, and MRCMap use the gene sets as subqueries, basing their modular score calculation on the unnormalized or normalized CMAP1 score. CMapCNA also calculates a modular score by projecting the reference profiles onto the singular value decomposition (SVD) first component associated to each gene set (called ‘module eigengene’, ME).

Most of these methods offer a way to interpret results on a larger scale, either by summarizing the scores or using a global selection process. GEMS2 could be used to calculate a summarized score by averaging the modular scores [47]. In contrast, FMCM and MRCMap use their modular scores to classify reference profiles as either similar or opposite to the query, thus identifying a minimal set of profiles that could induce the desired effect across all gene sets of interest. Similarly, CMapCNA selects the top-ranked profiles in the modules of interest or looks for similar effects using clustering over the ME projections.

#### Global score

Before the score calculation, methods that do not propose a modular score, project either the reference profiles alone or jointly with the query signature. MNBDR projects the reference profiles from the gene feature space to the gene set feature space, calculating the importance score associated with each gene set. GLHSC and KNeMAP project both the query signature(s) and the reference profiles. GLHSC calculates the GSEA enrichment score for each identified gene set, while KNeMAP defines feature vectors (i.e. the number of differentially expressed genes (DEGs) present in each gene set).

GLHSC and KNeMAP use a correlation score, as a global metric to compare the transformed query signature and reference profiles, while MNBDR proposes its own global score.

### Methods using predefined gene sets

#### GEMS2

GEMS2-specific notations are listed in Table 3.

**Table 3:** GEMS2 notations.

	**Original notation**	**Reconciled notation**
Modular similarity score between the reference profile r and the i-th gene set.	S	${gems}_{r,i}$
Global similarity score associated to the reference profile r.	-	${GEMS}_{r,i}$
Subsets of up- and down-regulated genes in the query that belong to the i-th gene set.	-	${sx}_i^{+},{sx}_i^{-}$
Enrichment score for up-regulated gene set subquery and down-regulated gene set subquery.	${KS}_{up},{KS}_{down}$	${ES}_{up},{ES}_{down}$
Number of genes in ${sx}_i^{+}$ and ${sx}_i^{-}$subsets.	n	${N}_{sx_i^{+}},{N}_{sx_i^{-}}$
Rank of the gene g in the rank-ordered version of R, ranked from highest to lowest gene expression values.	V(j)	${r}_{ref}(g)$

#### Selection of the gene sets of interest

To find the gene sets that will be used as subqueries, GEMS2 performs an ORA over every set on the GeneSetDB. A hypergeometric test is run to determine if DEGs in the query signature (S_X_), are enriched in certain gene sets.

#### Scores calculation

##### Modular scores

GEMS2 follows the CMAP 1.0 [9] strategy and calculates a similarity score between each gene set subqueries (up- and down-regulated $\left({sx}_i^{+},{sx}_i^{-}\right)$) and each reference profile in the RefProfDB, using a signed one-sample KS statistic. The ES_up_ and ES_down_ enrichment scores are calculated as follows:


$$ {ES}_{up}=\left\{\begin{array}{@{}ll}a, & if\ a>b\\{}-b, & if\ b>a\end{array}\right. $$

Where,


$$ a=\overset{N_{{\mathrm{sx}}_i^{+}}}{\underset{j=1}{\max }}\left[\frac{j}{N_{{\mathrm{sx}}_i^{+}}}-\frac{r_{ref}\left({\mathrm{sx}}_i^{+}(j)\right)}{N_R}\right] $$

$$ b=\overset{N_{{\mathrm{sx}}_i^{+}}}{\underset{j=1}{\max }}\left[\frac{r_{ref}\left({\mathrm{sx}}_i^{+}(j)\right)}{N_R}-\frac{\left(j-1\right)}{N_{{\mathrm{sx}}_i^{+}}}\right] $$

ES_down_ is calculated in the same manner with the subset of genes${sx}_i^{-}$.

Finally, the score ${gems}_{r,i}$ is calculated as follows:


$$ {gems}_{r,i}=\left\{\begin{array}{@{}ll}{ES}_{up}-{ES}_{down},& if\ \mathit{\operatorname{sign}}\left({ES}_{up}\right)\ne \mathit{\operatorname{sign}}\left({ES}_{down}\right)\\{}0,& otherwise\end{array}\right. $$

##### Summarized score

Reference profiles can be ranked based on the number of gene sets where they have a similar (${gems}_{r,i}>0$) or reverse (${gems}_{r,i}<0$) effect compared to the query. Additionally, scores can be summarized and visualized in a similarity matrix, with columns being the references and rows representing the enriched gene sets in the query.

Yu et al. [47] proposed a method based on KEGG [19] database, with the same strategy than GEMS2. In their paper a global score was calculated over the k reference replicates and the total number of enriched gene sets in the query:


$$ {GEMS}_r=\frac{1}{k}\sum_{\omega =1}^k\sum_{i=1}^n{gems}_{r_{\omega },i} $$

### GSLHC

#### Selection of the gene sets of interest

In GSLHC, the query may be a single signature, or a group of signatures associated with perturbagens that have known shared properties. Enriched gene sets in the query are determined using GSEA. The resulting enrichment scores are stored in the fS_x_ matrix, with each column being a sample and each row corresponding to a gene set enriched across every instance of the query.

#### Data projection

Before the score calculation, GSLHC projects all the RefProfDB reference profiles from the gene feature space to the gene set feature space by calculating the GSEA enrichment score for each gene set in the GeneSetDB. These scores are stored in the functional reference profiles matrix F, where each column is a reference profile, and each row corresponds to a gene set.

#### Scores calculation

##### Global score

The rows on the functional reference profiles matrix are filtered (F_x_) to keep only those included in fSx, i.e. enriched gene sets in the query.

GSLHC uses $\rho$ to compare the reference profiles and the query signatures. Profiles can then be ranked based on this score, and the results can be visualized using a two-way hierarchical clustering over a matrix where F_x_ and fS_X_ are merged, with correlation distance as metric.

### Cogena

#### Selection of the gene sets of interest

##### From the query signature

Cogena employs one of several clustering methods available in the workflow [35] to identify clusters of co-expressed DEGs based on their expression across query state replicates. These clusters can then be annotated using an ORA (e.g. hypergeometric test) over each set in the GeneSetDB.

##### From the reference profiles

From each reference profile the sets of top N $\left(N\in{\mathbb{N}}^{+}\right)$ up- and down-regulated genes are selected.

#### Scores calculation

##### Modular scores

To calculate the modular score of Cogena, a hypergeometric test is run between each reference gene set and the query co-expressed gene sets. For identifying perturbagens with opposite effects, clusters of up-regulated query genes are compared against down-regulated reference gene sets, and vice versa. To identify similar effects, clusters of up- and down-regulated query genes are compared to up- and down-regulated reference gene sets, respectively. The score is the negative log2 false discovery rate of the test.

### Methods using network inferred gene sets

#### FMCM

FMCM-specific notations are listed in Table 4.

**Table 4:** FMCM notations.

	**Original notation**	**Reconciled notation**
Modular similarity score between each reference profile and the i-th gene module.	-	${fmcm}_{r,i}$
Modular similarity score; normalized across all reference profiles and the i-th gene module.	ES	${fmcm}_{r,i}^{\ast }$

#### Network construction and gene modules identification

FMCM takes a protein–protein interaction network (PPIN) as input and constructs a gene–gene interaction network (GGIN) and a function–function network (FFN) for each condition associated with the query-state: control (i.e. mock) and perturbagen (e.g. disease).

#### Construction of the gene–gene interaction network

For each condition, the pair of genes (g_1_, g_2_) is added into the GGIN if: (i) the correlation $\rho$ between their expression vectors (condition replicates) has a permutation p-value under a certain threshold and (ii) the pair of proteins they encode is linked on the PPIN.

##### Construction of the function–function network

Each GGIN is reduced to an FFN, where nodes correspond to enriched GeneSetDB terms, called functional modules. These modules are identified using a hypergeometric test on the genes within the GGIN. The edges represent inter-genes interactions between modules and the weights their number.

##### Selection of the genes of interest

A list of genes of interest associated with the query perturbagen condition is identified using a procedure detailed in Supplementary Section S1. The union of all functional modules (control and perturbagen) is used to query the RefProfDB. For each functional module, only the genes of interest are included in the subqueries.

#### Scores calculation

##### Modular scores

The FMCM modular score, ${fmcm}_{r,i}$, is calculated as described for ${gems}_{r,i}$ inGEMS2. The normalized version is as follows:


$$ {fmcm}_{r,i}^{\ast }=\left\{\begin{array}{@{}ll}\frac{fmcm_{r,i}}{\max_r\left({fmcm}_{r,i}\right)},& if\ {fmcm}_{r,i}>0\\{}\frac{-{fmcm}_{r,i}}{\min_r\left({fmcm}_{r,i}\right)},& if\ {fmcm}_{r,i}<0\end{array}\right. $$

##### Global selection process

For each functional module, reference profiles are classified depending on the ${fmcm}_{r,i}^{\ast }$ value indicating either an opposite (${fmcm}_{r,i}^{\ast }$ < −0.5) or similar (${fmcm}_{r,i}^{\ast }$ > 0.3) effect compared to the query perturbagen. A bipartite graph, see Fig. *2*B, named association map, can be constructed with nodes being functional modules and reference perturbagens and edges indicating opposite or similar effects. Depending on the application, a minimal set of reference perturbagens with purely opposite or similar effect can be identified to cover all functions.

For example, if the query state of interest is associated to a disease, the association map will help to determine the minimum set of purely beneficial molecules (${fmcm}_{r,i}^{\ast }$ < −0.5) that cover all the functions associated with the pathological state.

### MRCMap

MRCMap-specific notations are listed in Table 5.

**Table 5:** MRCMap notations.

	**Original notation**	**Reconciled notation**
Modular similarity score between each reference profile and i-th regulatory unit.	-	${mrcm}_{r,i}$
Modular similarity score; normalized across all reference profiles and the i-th regulatory unit.	-	${mrcm}_{r,i}^{\ast }$

#### Network construction and gene modules identification

The Transcriptional Network Inference (TNI) pipeline [48] has three steps, detailed in the Supplementary Section S2. Gene modules in the network, named regulons, correspond to the set of target genes regulated by a specific transcription factor (TF), meaning that all genes within a module share a common regulatory mechanism controlled by the same TF.

#### Selection of the gene sets of interest

MRCMap determines enriched TF regulons within the network by analyzing the DEGs from the query signature, using enrichment analysis methods like GSEA.

#### Scores calculation

##### Modular score

The MRCMap modular score, ${mrcm}_{r,i}$, and its normalized version ${mrcm}_{r,i}^{\ast }$, are calculated as described in FMCM.

##### Global selection process

The same strategy described on FMCM, is used by MRCMap, to classify references having an opposite (${MRCM}_{r,i}$ < 0) or similar (${MRCM}_{r,i}$ > 0) effect compared to the query perturbagen and then selecting a minimal set of them.

### CMapCNA

CMapCNA-specific notations are listed in Table 6.

**Table 6:** CMapCNA notations.

	**Original notation**	**Reconciled notation**
First principal component (module eigengene) of the i-th gene module.	-	${\gamma}_i$
Projection of the reference profile r into the module eigengene associated to i-th gene module.	ME	${ME}_{r,i}$

#### Network construction and gene modules identification

CMapCNA employs the Weighted Correlation Network Analysis (WGCNA) [49] to identify co-expressed genes modules based on their expression signatures across query state replicates (see Supplementary Section S3 for details).

Subsequently, SVD is performed on each gene module, with ${\gamma}_i$ representing the first principal component (ME) of the i-th gene module.

#### Scores calculation

##### Modular scores

For each reference and each gene module, the score corresponds to the projection of the reference profile onto the ME of the module:


$$ {ME}_{r,i}={v}_{ref}\left(R\cap{gm}_i\right)\bullet{\gamma}_i $$

##### Global selection process

If the query corresponds to a disease signature and the reference to drug profile, molecules with highest or lowest modular scores generally have a therapeutic effect. Alternatively, if the aim is to find perturbagens with similar effects, the profiles can be clustered based on the modular scores.

### MNBDR

MNDBR-specific notations are listed in Table 7.

**Table 7:** MNBDR notations.

	**Original notation**	**Reconciled notation**
Importance score of the i-th gene module in the query signature.	$Imp$	${Imp}_{q,i}$
Importance score of the i-th gene module in the reference profile r.	*V(i)*	${Imp}_{r,i}$
Minimum and maximum differential expression values of all the genes in the query that belong to the i-th gene module.	$Fmin, Fmax$	${Fmin}_{q,i},{Fmax}_{q,i}$
Minimum and maximum differential expression values of all the genes in the reference that belong to the i-th gene module.	$Fmin, Fmax$	${Fmin}_{r,i},{Fmax}_{r,i}$
Vector of PageRank scores associated with the query gene modules at the k-th iteration.	${P}_k$	${P}_k$
Position of the i-th module in the ranked list of importance modules in the reference profile r.	*P(i)*	${\omega}_r(i)$
Global similarity score between the query and the reference r.	*S*	${MNBDR}_r$

#### Network construction and gene modules identification

MNBDR constructs a module network (MN) with nodes being gene modules identified by clustering from a PPIN, and edges being their cross-talks (see Supplementary Section S4 for details).

#### Selection of the gene sets of interest

##### Query projection for prior information extraction

For each one of the n modules in MN, the importance of the i-th gene module is calculated as follows:


$${Imp}_{q,i}=\left\{\begin{array}{@{}ll}{Fmax}_{q,i}-{Fmin}_{q,i},& if\ {Fmax}_{q,i}>0\ and\ {Fmin}_{q,i}<0\\{}\max \left(\left|{Fmax}_{q,i}\right|,\left|{Fmin}_{q,i}\right| \right),& otherwise\end{array}\right.$$

With ${Fmin}_{q,i}=\min \Big(\Delta{v}_{qry}(S\cap{gm}_i)\Big)$ and ${Fmax}_{q,i}=\max \Big( \Delta{v}_{qry}\left(S\cap{gm}_i\right) \Big)$.

The vector $\left[{Imp}_{q,1},\dots, {Imp}_{q,n}\right]$ characterizes the difference of gene expression levels within the query signature for each module [39].

##### Random walk algorithm

Once the query is projected onto the gene module’s feature space, a network propagation algorithm (Fig. *2*D) is run to simulate the cross-talks across gene modules and infer the PageRank [50] scores associated to each module. The vector ${P}_k\in{\mathbb{R}}^n$ is the PageRank scores at the k-th iteration and is defined as follows: While 


$$ {\left\Vert{P}_{k-1}-{P}_k\right\Vert}_2>d, $$

$$ {P}_k=\lambda W{P}_{k-1}+\left(1-\lambda \right){P}_0 $$

With,

- The transition matrix $W=\left({w}_{ij}\right)$ and ${w}_{ij}=\frac{a_{ij}}{\sum_k{a}_{kj}}$;
- The prior information about the modules ${P}_0=\left[{Imp}_{q,1},\dots,\right. \left. {Imp}_{q,n}\right]$;
- The damping factor $\lambda\ \left(0<\lambda <1\right)$ that avoids that the information spreads out over the whole network, hiding the local neighborhood of the important nodes. Typically, in PageRank $\lambda =0.85$.

The final PageRank scores are used to rank the gene modules in descending order, only the top m will be selected.

#### Data projection

As with the query signature, the reference profiles signatures in the RefProfDB are projected from the gene’s feature space to the module’s feature space by calculating the importance score of each gene module in each reference profile, as follows:


$${Imp}_{r,i}=\left\{\begin{array}{c}{Fmax}_{r,i}-{Fmin}_{r,i}, if\ {Fmax}_{r,i}>0\ and\ {Fmin}_{r,i}<0\\{}\max \left(\left| {Fmax}_{r,i}\right|,\left|{Fmin}_{r,i}\right| \right), otherwise\end{array}\right.$$

With ${Fmin}_{r,i}=\min \left(\Delta{v}_{ref}\left(R\cap{gm}_i\right)\right)$ and ${Fmax}_{r,i}=\max \Big(\Delta{v}_{ref}\left(R\cap{gm}_i\right)\Big)$.

#### Scores calculation

##### Global score

The score associated with the reference perturbagen r is:


$$ {\mathrm{MNBDR}}_r=\sum_{i=1}^m\frac{Imp_{r,i}}{\left|{\omega}_r(i)-i\right|+1} $$

With, ${\omega}_r(i)$ the position of the ith module in the ranked module list in perturbagen r response. If the top m modules in the query are also important in the reference profile their MNBDR score will be high.

### KNeMAP

#### Network construction and gene modules identification

KNeMAP constructs a prior knowledge network from heterogenous data sources, being gene–gene edges (e.g. PPIN, GGIN) or gene-entity edges (e.g. gene-disease, gene-pathway). First, they are transformed into individual gene–gene networks and unified into a comprehensive prior knowledge network (see Supplementary Section S5 for details). Then, gene modules are identified using an agglomerative hierarchical clustering algorithm.

#### Data projection

KNeMAP compares the query and the reference perturbagen-induced transcriptomic alterations using two feature vectors, ${FV}_q\in{\mathbb{R}}^n$ and ${FV}_r\in{\mathbb{R}}^n$, respectively. These capture the fraction of DEGs within each gene module, and are defined as follows:


$$ {FV}_q={\left[\frac{\mid{S}_X\cap{gm}_i\mid }{N_{S_X}}\right]}_{i\in [\![ 1,n]\!] }\ \mathrm{and}\ {FV}_r={\left[\frac{\mid{R}_X\cap{gm}_i\mid }{N_{R_X}}\right]}_{i\in [\![ 1,n]\!] } $$

#### Scores calculation

##### Global score

KNeMAP features vectors are then compared using a similarity metric as $\rho$ or the cosine similarity, and the reference profiles can then be ranked based on the result.

## Network driven methods

The common ground of network-driven methods lies in the usage of network biology concepts and algorithms, as shown in Fig. *2*. However, they lack a common workflow that would enable the unification and generalization of their structure. Therefore, each method is presented independently but with reconciled notations.

The MFM method [41], relies on a gene–gene network to evaluate the mutual predictability between the disease-associated genes and the DEGs in the molecules profiles. Jambada et Shin [44] proposed PNPDR, which constructs a bipartite network to identify drugs that could modulate disease-associated pathways through a network-propagation algorithm. Finally, both DDN [42] and DRIE [43] leverage KEGG signaling pathways and incorporate gene interaction types in their score calculations. DDN does this by transforming the perturbagens signatures with an impact factor, while DRIE introduces an inhibition score using the shortest path in the disease-specific network.

### MFM

MFM-specific notations are listed in Table 8.

**Table 8:** MFM notations.

	**Original notation**	**Reconciled notation**
Threshold for filtering the up- and down-regulated genes in the query.	*m*	$\alpha$
Threshold for selecting the up- and down-regulated genes in the reference.	*k*	$\beta$
Mutation-associated deregulated genes in the query.	*MAG*	${S}_X$
Subset of deregulated genes in the reference profile.	*DRG*	${R}_X$
Area under the ROC curve inferred from the mutual predictability scores between gene sets A and B.	${AUC}_{A-B}$	${AUC}_{A\to B}$
Global similarity score between the query and the reference r.	*MP*	${MFM}_r$

#### Network construction

MFM, uses an external functional linkage network (FLN) constructed by the integration of different functional genomics features using a naïve Bayes classifier [51]. The FLN is defined as $F=\left(V,E,W\right)$, with V the set of genes, E the set of functional links between them and W the weights of those links.

#### Query genes selection

Known mutated driver genes associated with the query perturbagen state (K) are mapped into the $F,$where $K\subseteq V$. Then the first nearest neighbors (1-NN) (and eventually the second nearest neighbors 2-NN) of the driver genes are selected. The edges among the remaining genes with weights above $\omega$ are kept, in order to remove weaker links. The filtered graph obtained is ${F}^{\prime }=\left({V}^{\prime },{E}^{\prime}\right)$ such as, for two genes u and v $\left(u\in V,v\in V\right)$: ${E}^{\prime }=\Big\{\left\{u,v\right\}\in E\ \mid{w}_{uv}>\omega \Big\}$, with ${w}_{uv}\in W$ the weight of the edge between u and v.

The remaining set of genes (V′) is further filtered. Genes are retained if (i) their expression levels exceed a specific threshold and (ii) they belong to an enriched pathway identified by PWEA [52], a topology-based enrichment analysis. Finally, the top $\alpha$ up- and down-regulated genes are selected as (mutation-associated) query genes (${S}_X$).

#### Reference genes selection

The top $\beta$ up- and down-regulated genes in each reference profile are selected (${R}_X$).

#### Functional score calculation

MFM uses mutual predictability to quantify the correlation between the selected sets of genes ${S}_X$ and ${R}_X$ (see Supplementary Section S6 for details). The final score is defined as the geometric mean of AUCs, which characterizes the predictability between the two gene sets:


$$ {MFM}_r=\sqrt{AUC_{R_X\to{S}_X}\times{AUC}_{S_X\to{R}_X}} $$

The higher the MFM score, the stronger the functional relationship between the reference and the query, making it a candidate for further investigation.

### PNPDR

PNPDR-specific notations are listed in Table 9.

**Table 9:** PNPDR notations.

	**Original notation**	**Reconciled notation**
Bipartite graph.	*G*	*B*
Node associated to a functional pathway.	*-*	*u*
Node associated to a reference profile.	*-*	*v*
Final labels for the reference profile v and the functional pathway u.	*f*	${l}_f(v),{l}_f(u)$
Initial labels for the reference profile v and the functional pathway u.	*l*	${l}_0(v),{l}_0(u)$
Global similarity score between the query and the reference r.	*Z-score*	${PNPDR}_r$

#### Network construction

##### Query pathways identification

First, GSEA is performed on each query state signature replicate to identify the query-specific pathways, i.e. the union of the enriched pathways from the GeneSetDB.

##### Pathway-reference association

Then, another GSEA is performed on each reference profile in RefProfDB. The resulting enrichment scores are stored in the reference pathway enrichment matrix, where each column is a reference profile, and each row corresponds to a pathway in the GeneSetDB.

##### Pathway-reference network construction

###### Label initialization

Prior knowledge about the reference profiles and pathways is used to initialize the bipartite graph $B=\left(U,V,E,W\right)$ whit: $U$ the set of nodes corresponding to the pathways in the GeneSetDB, $V$ the set of nodes corresponding to the references, $E$ the set of edges between those nodes ($U$ and $V$) and $W=\left({w}_{vu}\right)$ the matrix containing their weights (that correspond to the previously mentioned pathway enrichment matrix).

The enriched pathways in the query ($\subseteq U$) are labeled as ${l}_0(u)=1$, others as ${l}_0(u)=0$. Similarly, labels associated with the references ($\subseteq V$) previously known for interacting with the query state are initialized to ${l}_0(v)=1$ and others to ${l}_0(v)=0$.

###### Network propagation

Based on the hypothesis that references with similar responses should target the same pathways, PNPDR uses a semi-supervised network propagation strategy (Fig. *2*D).

First, the weights of the bipartite graph are normalized, the matrix of normalized weights $\left({W}^{\ast}\right)$ is defined as:


$$ {W}^{\ast }={D}_v^{-1/2}\ast W\ast{D}_u^{-1/2} $$

With ${D}_v$ and ${D}_u$ the diagonal matrixes holding the sum of all weights for each node.

Then, the final pathways ($l(u)$) and references ($l(v)$) labels scores are determined. Iteratively the labels are calculated until the learning process is completed, and they converge. The formula associated with the labels at the k-th iteration are:


$$ \mathrm{For}\ \mathrm{each}\ \mathrm{v}\in \mathrm{V},l{(v)}^k=\left(1-\alpha \right){l}_0(v)+\alpha \sum_{u\in U}{W^{\ast}}_{i_v,{i}_u}l{(u)}^{k-1} $$

$$ \mathrm{For}\ \mathrm{each}\ \mathrm{u}\in \mathrm{U},l{(u)}^k=\left(1-\alpha \right){l}_0(u)+\alpha \sum_{v\in V}{W^{\ast}}_{i_v,{i}_u}l{(v)}^{k-1} $$

With $\alpha \in \left[0,1\right]$ a parameter that regulates the relative weight of the initial label and the learned label.

#### Scores calculation

The values of the final references’ labels can then be normalized to obtain a PNPDR score:


$$ {PNPDR}_r=\frac{l_f(r)-{\mu}_l}{\sigma_l} $$

With ${\mu}_l$ and ${\sigma}_l$ the mean and respectively the standard deviation of the references labels scores distribution.

Finally, the associated p-value can be estimated using the Gaussian distribution, and references can be ranked based on the PNPDR score. Top-ranked references can then be considered as potential candidates for repurposing.

### D‌DN

DDN-specific notations are listed in Table 10.

**Table 10:** DDN notations.

	**Original notation**	**Reconciled notation**
Query perturbation signature.	*-*	S*
Reference perturbation signature.	*-*	R*
Perturbation factor of a gene g in the query perturbation signature.	*PF(g)*	S*(g)
Perturbation factor of a gene g in the reference perturbation signature.	*PF(g)*	R*(g)
Subset of upstream genes of g.	-	${u}_g$
Number of downstream genes of g.	${N}_{ds}(g)$	${N}_{ds}(g)$
Binary indicator representing the type of interaction between the genes g and h.	${\beta}_{gh}$	${\lambda}_{gh}$
Global similarity score (i.e. inhibition score) between the query and the reference r.	*DDN*	${DDN}_r$

#### Network construction

##### Global network construction

A global network is constructed from the graphs that model each one of the human signaling pathways in KEGG pathways database [19]. In each graph, nodes are genes and edges are the distinct types of interactions (activation, inhibition, …) between them (Fig. *2*C). ROntoTools package [53] is used to calculate the union all KEGG signaling pathways, all the adjacency matrices representing the pathways are then merged in a unified adjacency matrix, that represents the global network.

##### Subgraph extraction

The known query-associated genes, and the reference-associated ones form the nodes of the reference-query subgraph (RQN). Only the edges that are the shortest paths connecting the two gene sets are kept in the subgraph.

#### Scores calculation

First an impact analysis [54], is performed on the RQN subgraph to determine the impact caused by a perturbagen on the genes that are specific to the condition of interest [42]. The perturbation factor for each query-associated gene g if calculated as follows:


$$ {S}^{\ast }(g)=\Delta{v}_{qry}(g)+\sum_{h\in{u}_g}{\lambda}_{gh}\frac{S^{\ast }(h)}{N_{ds}(h)} $$

With ${u}_g$ the set of upstream genes of g, ${N}_{ds}(h)$ the number of downstream genes of h and ${\lambda}_{gh}$ the indicator representing the type of interactions between the genes g and h: ${\lambda}_{gh}=1$ for activation and induction and ${\lambda}_{gh}=-1$ for inhibition and repression. The sets of upstream and downstream genes are provided by the KEGG database. For the genes that doesn’t have upstream genes the perturbation factor is equal to $\Delta{v}_{qry}(g)$.

The same process is done to infer the perturbation factor for each reference:


$$ {R}^{\ast }(g)=\Delta{v}_{ref}(g)+\sum_{h\in{u}_g}{\lambda}_{gh}\frac{R^{\ast }(h)}{N_{ds}(h)} $$

Finally, for a query - reference pair the DDN score is calculated as follows:


$$ {DDN}_r=\rho \left({S}^{\ast },{R}^{\ast}\right) $$

A high positive DDN indicates that the pair of perturbagens cause similar effects in the system. Conversely, a high negative score indicates an opposite effect.

### DRIE

DRIE-specific notations are listed in Table 11.

**Table 11:** DRIE notations.

	**Original notation**	**Reconciled notation**
Effect score of the reference r on the query-associated gene g.	*E*	${E}_r(g)$
Set of edges composing the shortest path between the genes g and h.	${L}_{gh}$	${L}_{gh}$
Accumulation of the interaction between the reference-perturbed gene g and the query-associated gene h.	${\beta}_{gh}$	${\beta}_{gh}$
Binary indicator representing the type of interaction characterized by the k-th edge in the shortest path ${L}_{gh}$.	${\beta}_k$	${\lambda}_k$
Global similarity score (i.e. inhibition score) between the query and the reference r.	*S*	${DRIE}_r$

#### Network construction

##### Query-specific network

DRIE uses similar strategy as DDN [42] for the network construction. However, instead of constructing a global network from all the KEGG pathways, it only combines the pathways associated with the query perturbagen state to construct a query-specific network.

##### Shortest path inference

DEGs from the query signature (S_X_) and the reference profile that is being evaluated (R_X_) are then mapped into the query-specific network, where all the interactions between reference-associated genes and query-associated genes are modeled. The shortest path ${L}_{gh}$ between a pair of genes (g,h) is then be inferred using the reference gene g as source and query gene h as destination (see Fig. *2*C).

#### Scores calculation

The functional score calculated by DRIE is an inhibition score, based on the hypothesis that reference-associated DEGs should have an effect opposite to the query ones to be potential treatment candidates.


$$ {DRIE}_r=\sum_{h\in{S}_X}-{E}_r(h)\times \operatorname{sign}\left(\Delta{v}_{qry}(h)\right) $$

With E_r_(g) the effect score of the reference r over the query-associated gene g.


$$ {E}_r(h)=\operatorname{sign}\left(\sum_{g\in{R}_X}\frac{\beta_{gh}\times \Delta{v}_{ref}(g)}{L_{gh}+1}\right),\mathrm{where}\ {\beta}_{gh}=\prod_{k=1}^{L_{gh}}{\lambda}_k $$

${\beta}_{gh}$ represents the accumulation of the interaction between the reference perturbed gene g and the query-associated gene h and ${\lambda}_k$ is the indicator being the type of interactions between the genes in the ${L}_{gh}$ path. If there is an activation ${\lambda}_k=1$, if there is an inhibition ${\lambda}_k=-1$.

## Discussion

In this study, we reconciled twelve methods for omics signature comparison applied to drug repurposing. These methods address a significant limitation of traditional approaches that rely solely on gene expression signatures without considering available knowledge in systems biology. Each method presents a different strategy, but some similarities were found in their approaches even if they are not built upon each other. Therefore, we classified them into two main categories: gene set-driven methods and network-driven methods (Fig. 1).

On one side, gene set-driven methods utilize predefined or network-inferred genes sets to draw functional relations between perturbagens. Cogena extracts these from both query signatures and references profiles, comparing them via ORA. While other approaches use gene sets as subqueries to calculate modular scores or as dimensions of a new feature space for projecting gene expression data. GEMS2, FMCM and MRCMap apply the ‘subquery strategy’ but differ in gene sets identification and selection. GEMS2 uses predefined gene sets, while FMCM and MRCMap use PPI and TF-centered networks, respectively, to extract the gene sets of interest. Instead, CMapCNA, GSLHC, KNeMAP, and MNBDR are based on the ‘data projection strategy’. CMapCNA calculates a modular score by projecting reference profiles onto the principal component of each gene set. While GSLHC, KNeMAP, and MNBDR project both perturbagen signatures onto a new feature space using all the selected gene sets simultaneously and calculate a similarity score. Therefore, no modular scores are obtained.

**Figure 3:**
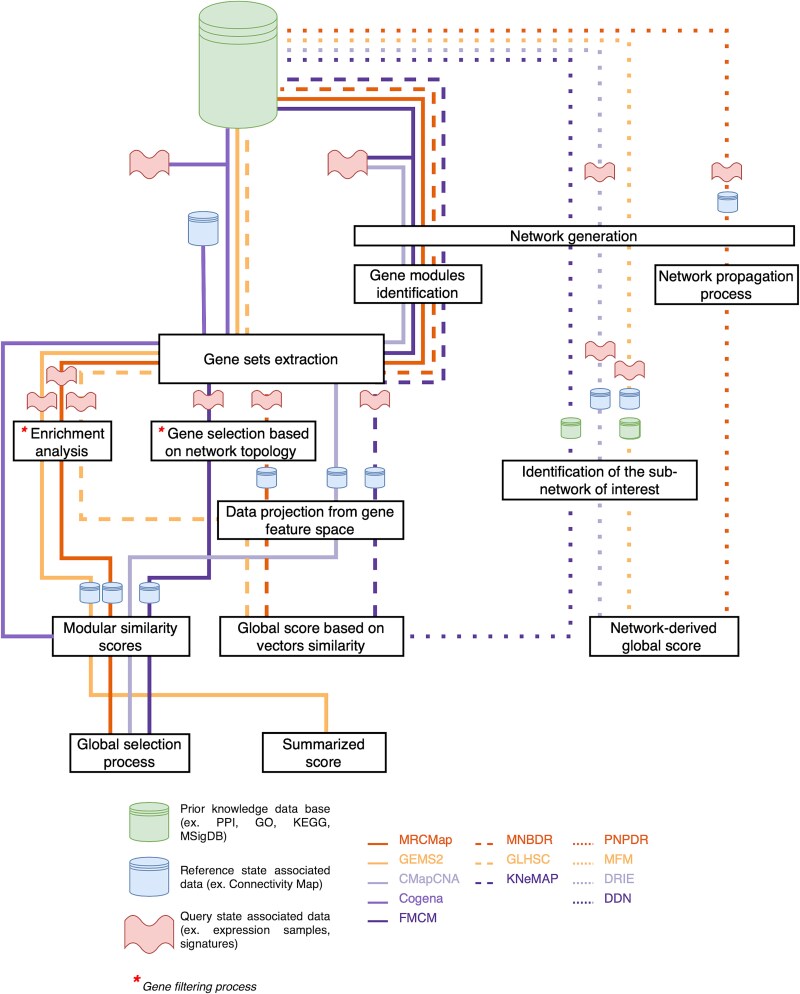
Unified representation of the stages in system biology-based connectivity scores. This schema is a unified representation that illustrates the common workflow stages in the studied methods as well as the methodological variations. Each line represents the specific path followed by each method, highlighting the similarities and differences between the approaches.

After dissecting gene set driven methods and standardizing their common workflow (Fig. 3) we can easily highlight their similarities and facilitate the identification of potential pain points for future developments. For example, several gene co-expression module identification algorithms have been proposed based on WGCNA [55–57]. Recently, Zhang et al. [58] published a method integrating both functional and gene expression similarities for gene module identification. These new methodologies could be used as the first step of the workflow.

On the other side, network-based approaches are more diverse, using network topology and prior knowledge. MFM [46] relies on a functional linkage network and integrates both gene mutation data and gene expression profiles. This method is expected to be effective when known mutated genes are present in the query state. PNPDR [43] can integrate diverse types of omics data and prior knowledge to constitute a bipartite graph that is used to label the nodes through network propagation. While DDN [42] and DRIE [43] use signaling pathway networks to elucidate the complex interactions between perturbagens. A common structure between these methods is harder to elucidate, nevertheless, concepts and algorithms in network biology are shared points in their global structures (Fig. 3). Future developments shall not stop at the list of network biology algorithms listed here. Indeed there are many network based approaches, like network alignment methods [59], that could be exploited by network-based. Particularly, local alignments could be interesting to explore sub-networks associated with specific gene modules [60].

Even if the methods are not built upon each other, the chronology reveals an evolving trend over the years (Fig. 1). Predefined gene sets are replaced by data-driven and knowledge enriched biological networks. There is a growing trend to integrate more specific data and leverage increasing prior information. Deep learning approaches are entering the field, offering new strategies for integrating heterogeneous data, e.g., through convolutional networks to identify new drug-disease associations [61, 62] and representation learning [63], though those methods focus only on available knowledge and are not driven by omics signature comparisons. The quality of the data is essential for systems-biology-based approaches, since their scoring strategy is heavily influenced by it. These criteria, along with application aims, should be considered before selecting a method.

Table 12 provides an overall summary to help users and developers to choose the most suitable method for their needs, based on ease of use across several criteria. As described, use of systems biology can help integrate existing knowledge of gene-to-gene relationships into connectivity estimation. Moreover, some knowledge sources contain more a priori that others. To highlight this, we included an annotation indicating how sensible each method is to poorly described genes or genes rarely expressed (based on a theorical point of view of the methods).

**Table 12:** Comparative view of methods.

**Method name**	**Query**	**Integrated knowledge**	**Complex usage score**	**Sensitivity to rare genes**	**Modular score**	**Global score**	**Compared to**	**Case of study**	**Availability**	**Last updated**	**Resource link**
GEMS2	One gene expression profile	Predefined gene sets	1	low	yes	yes	1 (CMap1)	Valproic acid (VPA); hypoxia; breast cancer	Web UI	Broken link	http://www.biosino.org/GEMS2/
GSLHC	One gene expression profile	Predefined gene sets	2	low	no	yes	1 (CMap1)	Similar effects for 18 query compounds	Web UI	Broken link	http://cloudr.ncu.edu.tw/gslhc/
Cogena	multiple gene expression profiles	Predefined gene sets	1	low	yes	no	1 (CMap1)	Psoriasis	Package/ LGPL-3	November 2023	https://github.com/zhilongjia/cogena
FMCM	multiple gene expression profiles	PPIN from HPRD [64]^2^	4	very high	yes	global selection	1 (CMap1)	Colorectal cancer	no		no mention
MRCMap	One gene expression profile	Expression data for network inference	3	high	yes	global selection	1 (CMap1)	Bipolar disorder	no		codes available on request to the corresponding authors
CMapCNA	One gene expression profile	Predefined gene sets	4	low	yes	global selection	None	Breast cancer and non-alcoholic fatty liver disease	no		data available under reasonable to the corresponding authors
MNBDR	One gene expression profile	PPIN from String [65]^2^	4	low	no	yes	6 (similar and non-similar methods)	Breast cancer	Source/ Unlicensed	November 2020	https://github.com/nbnbhwyy/MNBDR
KNeMAP	One gene expression profile	Multiple (PPIN, GGIN, gene-pathway, etc.)	4	low	no	yes	3 (non-similar methods)	Drug exposure and exposure to nanomaterials	Source/ GPL-3	October 2024	https://github.com/fhaive/KNeMAP
MFM	Mutated genes from OMIM	Functional Network	3	low	no	yes	2 (non-similar methods)	Breast cancer, prostate cancer and leukemia	no		no mention
PNPDR	multiple gene expression profiles	Predefined Pathways + Known drug-disease interactions	5	medium	no	yes	None	Breast cancer	no		no mention
DDN	One gene expression profile	Signaling pathways	3	low	no	yes	1 (CMap1)	Pulmonary fibrosis, non-small cell lung cancer, prostate cancer, breast cancer	Package/GPL-3	October 2017	https://github.com/azampvd/DrugDiseaseNet
DRIE	One gene expression profile	Signaling pathways	3	low	no	yes	3 (similar and non-similar methods)	Colorectal cancer, breast cancer and lung cancer	Package/Unlicensed	July 2023	https://github.com/eshinesimida/DRIE

This table summarizes all the methods. ‘Query’ column precises the input requirements. ‘Integrated knowledge’ specifies the type of systems biology data used. ‘Complex usage score’ is an indicator of how complex it is to use the approaches, the higher the more complex; from 1 it’s given +1 for each of the following: network-based method, number of query profiles, need to build network from data, need to apply complex transformation to inputs, need to identify modules within a network. ‘Sensitivity to rare genes’ is the ability of a method to detect genes that are either poorly described or rarely expressed; this is enabled by building graph from experimental data and, if possible, query specific. ‘Modular score’ and ‘Global score’ column refers to the existence of such a score or at least a global selection or ranking process. ‘Compared to’ column refer to the number of methods against a method was benchmarked or compared. Other columns indicate the availability of the method.

In simple terms, sensibility is higher when relationships are directly inferred from experimental data (without a priori). For genes rarely expressed, it is necessary to increase the number of query conditions related to the phenotype of interest. This ensures that co-expression of phenotype-specific genes is captured. This is particularly relevant in FMCM and MRCMap.

Unfortunately, seven out of twelve of those methods are no longer available. Only five methods provide source code, yet none offer a containerized environment (e.g. Docker), which is regrettable given that many rely on outdated or unspecified software versions. For beginners in the field, methods such as GEMS2 or Cogena are preferable, as they are easier to implement and clearly explainable. This recommendation is based on simplicity (as shown in Table 12), not performance, since a comprehensive evaluation of these methods has yet to be conducted.

Indeed, to date, it is difficult to judge the performance of these twelve methods as they have not been systematically benchmarked in a unified manner. Some methods present application cases for specific diseases and confirm their treatment predictions via retrospective literature. Others use external databases (e.g. ATC levels, disease treatments) as gold standards to annotate the signature datasets and compare the new method’s performance against an existing one, usually CMAP, but rarely against other systems-biology-based connectivity scores. In some methods (GEMS2, Cogena, MRCMap), there are no proper evaluation metrics such as FDR, ROC, etc. and just a description of the qualitative differences. Although network-based approaches are highly referenced, it would be valuable to have benchmarking that limits data biases and enables systematic evaluation of current methods. Similar to the benchmarking done for machine learning-based methods using eleven evaluation databases [66] or for signature-based methods using simulated data [67].

Ultimately, it is valuable to contextualize these approaches within the current developments in artificial intelligence and deep learning in the field. A promising technique in deep learning leverages latent representations or embeddings to model expression profiles. On one hand, functional representations can be inferred by integrating data from systems biology databases [68]. On the other hand, such embeddings are already being used for in silico drug repurposing [69, 70]. Siamese neural network have also been applied to predict connectivity, although in previous work this was based on comparing latent representations of SMILES strings, rather than expression profiles [71]. From a broader perspective, LLMs agents [72] and Retrieval Augmented Generation (RAG) [73] could become increasingly valuable for identifying and collecting omics datasets from vast public repositories that best match a given phenotype of interest.

## Conclusion

In conclusion, we identified twelve systems biology connectivity scores from the literature and reconciled their formulations. This reconciliation revealed strong similarities between the different approaches, thereby simplifying further development in the field. We expect that this work will promote the progressive refinement of methodologies, preventing redundant efforts. Additionally, we aimed to generalize these methods to broaden their applicability across various data sources and different applications. Finally, the homogenization and categorization of these twelve scores can help their large-scale benchmarking, which we consider a fundamental element for their further democratization. Moreover, they can serve as a valuable state-of-the-art reference for future scores, including those employing new strategies such as deep learning.

## Supplementary Material

Supplementary_materials_bbaf387

## Footnotes

1 The names of these methods were given in this review, as the original papers did not provide names.

2 Note: can be generalized to other PPIN
