# Supplementary material for: Reconciling multiple connectivity-based systems biology methods for drug repurposing
Source: Brief Bioinform. 2025 Jul 30;26(4):bbaf387. doi: 10.1093/bib/bbaf387 (PMC12309248; doi:10.1093/bib/bbaf387)
Supplement: Supplementary_materials_bbaf387 [file supplementary_materials_bbaf387.docx]

# Supplementary

## Supplementary Section S1: FMCM selection of the genes of interest

To be considered as a gene of interest in the FMCM approach a gene has to: (i) be differentially expressed between the two conditions associated to the query state (control and perturbagen) and (ii) pass the two criteria of the GSTop filtering procedure:

1. The gene appears in at least ones in the control or perturbagen GGIN.
2. The gene’s clustering and degree coefficients increase or decreases along the state sequence, i.e. between the conditions control and perturbagen. The degree coefficient $\partial$ of a node corresponds to the number of edges connected to it. And the local clustering coefficient Q of a node quantifies the degree to which nodes’ neighbors are interconnected. It measures the proportion of the neighbors of a node that are also connected to each other divided by the number of possible links between them [1] the clustering coefficient is calculated as follows:

$$Q=\frac{2e}{\partial(\partial-1)}$$

With e the number of links among the neighbors of the node.

Note: This second criterion in more judicious in the cases where the state sequence has at least three states as in the original paper [2].

## Supplementary Section S2: Transcriptional Network Inference (TNI) Protocol

The transcription factor (TF) centered network is constructed using the RTN package [3] in R, which employs a reverse engineering algorithm [4] to derive the network structure by mapping the significant interactions between known TFs and all potential targets in the gene expression matrix, which contain several expression profiles.

1. First, mutual information (MI) [5] is computed between each regulator and all potential target genes to quantify the dependency between their expression profiles. The MI score between to vectors X and Y representing the expression profiles of two genes is:

$$MI\left( X;Y \right)=\sum_{x \in X} \sum_{y \in Y} p_{XY}\left( x,y \right)\times\text{log}\left( \frac{p_{XY}(x,y)}{p_{X}\left( x \right)p_{Y}(y)} \right)$$

Where $p_{XY}\left( x,y \right)=P(X=x,Y=y)$ is the joint probability mass function of X and Y, and $p_{X}\left( x \right)=P\left( X=x \right)$ and $p_{Y}\left( y \right)=P(Y=y)$ are the marginal probability mass function of X and Y, respectively.

Interactions where mutual information falls below a minimum threshold are eliminated through **permutation analysis**.

1. Then, unstable interactions are removed by bootstrapping, for each bootstrap sample, a transcriptional network is reconstructed, and the consensus network is derived by retaining only the interactions consistently observed across the resampled datasets. This results in a robust relevance network.
2. Finally, the ARACNe algorithm based on the Data Processing Inequality (DPI) theorem, is applied to remove the weakest interaction in any triplet formed by two TFs and a common target gene, preserving the dominant TF-target pair. This step substantially reduced the number of indirect regulatory relationships. It is the resulting DPI-filtered transcriptional network that is subsequently interrogated in the enrichment analysis.

## Supplementary Section S3: Network construction and module detection with WCGNA

Let $X\in\mathbb{R}^{N_{S}\times k}$ be the gene expression matrix, where $N_{S}$ represents the number of genes (or probes) in the signatures associated to the query state and k the number of samples. $X=\left( x_{gs} \right)_{g\in\left⟦ 1,N_{S} \right⟧, s\in\left⟦ 1,k \right⟧}$, with $x_{gs}$the expression value of gene g in the sample s.

The weighted adjacency matrix $A\in\left⟦ 1,N_{S} \right⟧^{2}$ associated with the network is defined as $A=\left( a_{gh} \right)_{(g,h)\in\left⟦ 1,N_{S} \right⟧^{2}}$,such as:

$$a_{gh}=p_{gh}^{\theta}$$

With $p_{gh}=\left| \rho\left( x_{g\cdot},x_{h\cdot} \right) \right|$ the absolute Pearson’s correlation coefficient between the expression vectors associated to the genes g and h, and $\theta$ the power parameter.

The weighted network associated to A is the transformed into a network of topological overlap (TO). The topological overlap is a co-expression measurement that considers not only the correlation of two genes with each other, but also the extent of their shared correlations across the weighted network. The TO matrix $O={(o_{gh})}_{(g,h)\in\left⟦ 1,N_{S} \right⟧^{2}}$ such as the topological overlap between two genes g and h is equal to:

$$o_{gh}=\frac{a_{gh}+\sum_{m=1}^{N_{s}} a_{gm}\cdot a_{hm}}{\text{min(}\partial_{g}\text{,}\partial_{h}\text{)-}a_{gh}}$$

Whit $\partial_{g}$ and $\partial_{h}$ the clustering degrees of the nodes representing genes g and h.

A hierarchical clustering is then performed based on the distance matrix $D={(d_{ij})}_{(i,j)\in\left⟦ 1,N_{S} \right⟧^{2}}$ derived from the *O*, such as $d_{ij}=1-o_{ij}$. Techniques like complete linkage or average linkage can be used to perform the clustering.

Gene modules are finally identified based on the clustering results, typically by cutting the dendrogram to ensure that the modules have a minimum size $t\mathbb{\in N}$. They can be characterized using an enrichment analysis based in the GeneSetDB annotations.

## Supplementary Section S4: MNBDR network construction and gene modules identification algorithm

In the module network (MN) constructed by MNBDR, nodes represent gene modules and edges represent the cross-talks between them.

First, gene modules are identified from a PPIN using the MCODE algorithm from Cytoscape [6,7]. The PPIN is modeled by a graph $P=(V,E)$ where V is the set of nodes (proteins or coding genes) and $E\subseteq V\times V$ is the set of edges (interactions). Gene modules are then defined as clusters with more than t nodes.

Then, edges are added to represent cross-talks between modules, as follows:

1. For each pair of modules ${gm}_{i}$ and ${gm}_{j}$ ((*i,j)* $\in\left⟦ 1,n \right⟧^{2})$, the set of edges $\left( E_{i,j} \right)$ between them is determined as:

$E_{i,j}=\left[ \left\{ a,b \right\}\in E \right| a\in{gm}_{i}(\in V), b\in{gm}_{j}(\in V)], with i\neq j$

The total number of edges between the pair of gene modules is then inferred and a permutation pvalue is determined using a random sampling process to generate a null distribution for the number of edges.

1. Cross-talks are considered between modules with a significant number of edges (pvalue under a specific threshold). The adjacency matrix $A$ representing the cross-talks between modules in the network is then defined as:

$$A= (a_{ij}), \text{and }a_{ij}=\left\{ \begin{aligned} 1, if pvalue< \text{p}_{0} \\ 0, otherwise \end{aligned} \right.$$

## Supplementary Section S5: KNeMap network construction

|  | **Notations** |
| --- | --- |
| Number of gene-gene association data sources. | $\alpha$ |
| Gene-gene network representing the k-th gene-gene association data $\left( k\in\left⟦ 1,\alpha\right⟧ \right).$ | $\Gamma_{k}$ |
| Unified network for the gene-gene association data. | $\Gamma_{uni}$ |
| Bipartite network representing the gene-entity association data source. | *B* |
| Number of entities in the B network. | $\beta$ |
| Total number of inferred gene-gene networks from the prior knowledge data sources. | $\tau$ |
| Gene-gene network inferred from the prior knowledge data sources (x=1,2,…,$\tau$) (y=1,2,…,$\tau$) | *GG_x_, GG_y_* |
| Jaccard distance matrix, simple matching coefficient and the percentage of shared edges distance matrix between two gene-gene networks | $d_{J},d_{SMC},d_{PSE}$ |
| Global distance matrix between two gene-gene networks | $D$ |

### Supplementary Section S5.1: Gene-gene association data

Given a set of $\alpha$ gene-gene association data sources, each one can be represented by a network $\Gamma_{k}=(V_{k},E_{k})$, with a set of nodes $V_{k}$ representing the genes and a set of edges $E_{k}$ representing the interactions between them. The unified network is then defined as:

$$\Gamma_{uni}=\left( V_{uni}=\bigcup_{k=1}^{\alpha} V_{k},E_{uni}=\bigcup_{k=1}^{\alpha} E_{k}, W \right)$$

With W set of weights assigned to each edge in the network, equal to the number of data sources supporting it. For each edge $e\in E_{uni}$:

$$w\left( e \right)=\sum_{k=1}^{\alpha} \delta_{k}(e)$$

With $\delta_{k}$ the indicator function for each network $\Gamma_{k}$such that:

$$\delta_{k}\left( e \right)=\left\{ \begin{aligned} 1, if e\in E_{k} \\ 0, if e\notin E_{k} \end{aligned} \right.$$

### Supplementary Section S5.2: Gene-entity association data

Gene-entity association data is also transformed into a gene-gene similarity network for unification.

Let define a bipartite network *B=(U, V, F)* with *U* a set of nodes representing the $\beta$ entities, *V* a set of nodes representing the genes, *F* the set of edges between them and *A* the associated adjacency matrix.

$$A= \left( a_{uv} \right), \text{and }a_{uv}=\left\{ \begin{aligned} 1, if \left\{ u,v \right\}\in F \\ 0, otherwise \end{aligned}, with \right.u\in U and v\in V$$

The gene-gene similarity network $\Lambda=(V,E)$, is defined as follows:

- V the set of nodes representing the genes.
- E the set of edges representing a common association of two genes with the same entity. For two nodes x and y from V:

$$\left\{ x,y \right\}\in E if \exists z\in U | \left\{ z,x \right\} \in F and \left\{ z,y \right\} \in F$$

- W the weights of the edges representing the number of shared entities between a pair of genes. For each edge $e\in E$ between two nodes x and y:

$$w\left( e \right)=\sum_{k=1}^{\beta} A_{k,x}\cdot A_{k,y}$$

### Supplementary Section S5.3: Prior network: hierarchical merge of the individual networks

Once a gene-gene network is created for each one of the prior knowledge data sources using one of the two strategies presented, their edge weights were scaled to be between 0 and 1, where a value close to 1 represents a strong association and a value close to 0 represents a weak one.

Three distance matrixes are computed based on: the Jaccard index, the simple matching coefficient (SMC) and the percentage of shared edges, defined as follows for two networks GG_x_=(V_x_,E_x_) and GG_y_=(V_y_,E_y_):

- Jaccard distance: $d_{J}=1-\frac{|E_{x}\cap E_{y}|}{|E_{x}\cup E_{y}|}$
- SMC distance: $d_{SMC}=1-\frac{M_{oo}+M_{11}}{M_{oo}+M_{o1}+M_{10}+M_{11}}$, considering a binary representation of the edges in each graph $M_{oo}$ and $M_{11}$ corresponding to the number of matching 0’s and 1’s between the two networks and $M_{o1}$ and $M_{10}$ the mismatches.
- The percentage of shared edges distance: $d_{PSE}=1-\frac{|E_{x}\cap E_{y}|}{\left| E_{x} \right|+|E_{y}|}$

The global distance matrix $D=\left( d_{xy} \right)\in{[0,1]}^{\tau\times\tau}$is then calculated, such that for the two networks GG_x_=(V_x_,E_x_)and GG_y_=(V_y_,E_y_): $d_{xy}=\frac{1}{3}(d_{J}+d_{SMC}+d_{PSE})$

A hierarchical clustering was then performed based on the matrix D (with the Ward linkage method). The networks within each cluster are merged are then with the following strategy:

1. Edge weights are scaled, by a scaling factor $\lambda$, within each network of the cluster such that the median edge weight across the networks in the cluster is identical.
2. The clustered networks are merged together by summing their scaled edges.

This scaling and merging processes are repeated until only one final network is obtained, whose weights are again scaled to be between 0 and 1. The prior network generated for the KNeMap paper is available at <https://doi.org/10.5281/zenodo.7334711>.

## Supplementary Section S6: Details on mutual predictability calculation

|  | **Original notation** | **Reconciled notation** |
| --- | --- | --- |
| Mutual predictability variable cutoffs. | S_i_ | $\varepsilon$ |
| Mutual predictability score between two gene sets A and B for a gene i, using genes in A as seeds. | MP_A-B_ | ${mp}_{A\to B,i}$ |

The mutual predictability (MP) between two gene sets measures the capacity to which genes in one set can be used to predict genes in the other, and vice versa [8].

Let consider two set of genes A and B. The genes $h\in A$ are used as seed genes to calculate the mutual predictability score between A and B, associated with a gene $g \in B$ as follows:

$${mp}_{A\to B, g}=\sum_{h\in A} w_{gh}$$

Where $w_{gh}$ are the weights of the edges between genes g and h in the FLN. The score is equal to zero ${mp}_{A\to B,g}=0$ if the gene g isn’t connected to any seed gene. Genes on the FLN are then ranked, and different cutoffs $\varepsilon$ are used to compute the true positive rate (TPR or sensitivity) and false discovery rate (FDR).

$$TPR= \frac{TP}{TP+FN}$$

$$FDR= \frac{FP}{TN+FP}$$

With:

- True positive (TP): the number of genes included on the set B with a mutual predictability score above the cutoff ${mp}_{A\to B,g}>\varepsilon$ and $g\in B$.
- False positive (FP): the number of genes non included on the set B with a mutual predictability score above the cutoff ${mp}_{A\to B,g}>\varepsilon$ and $g\notin B$.
- True negative (TN): the number of genes non included on the set B with a mutual predictability score below the cutoff ${mp}_{A\to B,g}<\varepsilon$ and $g\notin B$.
- False negative (FN): the number of genes included on the set B with a mutual predictability score below the cutoff ${mp}_{A\to B,g}<\varepsilon$ and $g\in B$.

The ROC curve is obtained by plotting the TPR against the FPR, calculated at various cutoff settings $\varepsilon\in\mathbb{R}^{+}$. Then, the area under this curve (AUC_A🡪B_) is determined.

Note :

Different thresholds $\alpha$ and $\beta$ can be tested to determine the number of DEGs included in *S_X_* and *R_X_* gene sets, respectively:

- $\alpha\in\{1000, 3000, 5000,\ldots, \frac{\left| V' \right|}{2}\}$
- $\beta\in\{\alpha-500, \alpha-400, \ldots,\alpha,\ldots,\alpha+400, \alpha+500 \}$.

For this reason, the calculated AUC scores can vary. In such cases, one strategy is to select the pair of parameters that maximize the AUC. It is important to note that the optimal $\alpha$ and $\beta$ values may differ for the up- and down- regulated subsets.

1. Barabási A-L, Oltvai ZN. Network biology: understanding the cell’s functional organization. *Nat Rev Genet* 2004;**5**:101–13.

2. Chung F-H, Lee HH-C, Lee H-C. ToP: A Trend-of-Disease-Progression Procedure Works Well for Identifying Cancer Genes from Multi-State Cohort Gene Expression Data for Human Colorectal Cancer. Emmert-Streib F (ed.). *PLoS ONE* 2013;**8**:e65683.

3. Fletcher MNC, Castro MAA, Wang X *et al.* Master regulators of FGFR2 signalling and breast cancer risk. *Nat Commun* 2013;**4**:2464.

4. Pfaffenseller B, Da Silva Magalhães PV, De Bastiani MA *et al.* Differential expression of transcriptional regulatory units in the prefrontal cortex of patients with bipolar disorder: potential role of early growth response gene 3. *Transl Psychiatry* 2016;**6**:e805–e805.

5. Cover TM, Thomas JA. *Elements of Information Theory*. 1st ed. Wiley, 2005.

6. Pruitt KD. RefSeq and LocusLink: NCBI gene-centered resources. *Nucleic Acids Research* 2001;**29**:137–40.

7. Shannon P, Markiel A, Ozier O *et al.* Cytoscape: A Software Environment for Integrated Models of Biomolecular Interaction Networks. *Genome Res* 2003;**13**:2498–504.

8. Linghu B, Snitkin ES, Hu Z *et al.* Genome-wide prioritization of disease genes and identification of disease-disease associations from an integrated human functional linkage network. *Genome Biology* 2009;**10**:R91.
